# Pentraxin-3 Serum Levels Are Associated with Disease Severity and Mortality in Patients with Systemic Inflammatory Response Syndrome

**DOI:** 10.1371/journal.pone.0073119

**Published:** 2013-09-09

**Authors:** Simone Bastrup-Birk, Mikkel-Ole Skjoedt, Lea Munthe-Fog, Jens J. Strom, Ying Jie Ma, Peter Garred

**Affiliations:** 1 Laboratory of Molecular Medicine, Department of Clinical Immunology, Rigshospitalet, Copenhagen University Hospital, Faculty of Health Sciences, University of Copenhagen, Copenhagen, Denmark; 2 Department of Anaesthesiology, Glostrup Hospital, Copenhagen University Hospital, Faculty of Health Sciences, University of Copenhagen, Copenhagen, Denmark, Copenhagen, Denmark; University of Cincinnati, United States of America

## Abstract

The long pentraxin-3 (PTX3) is a key component of the humoral arm of the innate immune system. PTX3 is produced locally in response to pro-inflammatory stimuli. To investigate PTX3 levels and its use as a biomarker in patients with systemic inflammation, we developed a solid-phase enzyme-linked immunosorbent assay based on novel anti-PTX3 monoclonal antibodies detecting PTX3 with high sensitivity. The assay was applied on 261 consecutive patients admitted to an intensive care unit prospectively monitored with the systemic inflammatory response syndrome (SIRS). 100 blood donors were included as controls. PTX3 levels were elevated in patients (median = 71.3 ng/ml) compared with the controls (median = 0 ng/ml) (Mann-Whitney, p<0.0001). ROC analysis showed that PTX3 levels were significantly specific (85.0%) and sensitive (89.1%) to discriminate between healthy controls and patients (area under the curve (AUC) 0.922 (95% CI 0.892 to 0.946, p<0.0001)). Higher levels of PTX3 were associated with the development of sepsis, severe sepsis and septic shock (p = 0.0001). The serum levels of PTX3 correlated significantly with SAPS2 score (Spearman's rho 0.28, p<0.0001). Patients with high levels of PTX3 at admission did have a higher 90 day mortality rate than patients with the 25% lowest levels (Cox regression analysis, hazard ratio 3.0, p = 0.0009). In conclusion, we have established a highly sensitive and robust assay for measurement of PTX3 and found that its serum concentrations correlated with disease severity and mortality in patients with SIRS and sepsis.

## Introduction

Pentraxins are a superfamily of pattern recognition molecules belonging to the humoral arm of the innate immunity. Pentraxin-3 (PTX3) is the prototypic long pentraxin whereas the classical acute-phase protein, C-reactive protein (CRP), and serum amyloid P component (SAP), belong to the short pentraxins. This division is based on the length of their primary structure. Besides from a signal peptide, the primary transcript of PTX3 consists of a classical pentraxin like C-terminal domain containing the pentraxin signature (HxCxS/TWxS, where x is any amino acid) and a unique N-terminal domain [1].

PTX3 adopts to a complex multimeric formation creating an octamer composed of two covalently linked tetramers. PTX3 contains a single N-glycosylation site at Asn220 in the C-terminal domain that is fully occupied by complex type oligosaccharides. The glycosylation state has been shown to affect the binding to different ligands and therefore suggested to influence the biological activity [2].

In contrast to the short pentraxins, PTX3 is highly conserved throughout evolution from arachnids to man. It represents a functional ancestor of antibodies as it recognises conserved microbial moieties and initiates the immune response in coordination with the cellular arm [3].

PTX3 is produced in response to proinflammatory stimuli including IL-1β, TNF-α, microbial moieties and toll-like receptor (TLR) engagement. Neutrophil granulocytes store PTX3 in specific granules while it is synthesised de novo in a variety of cells, though primarily myeloid dendritic cells and mononuclear phagocytes [4]. However, the source of PTX3 production or release depends on the kind of inflammatory stimulus [5].

PTX3 is hardly detectable in healthy subjects with a concentration ≤2 ng/ml [6]. Under inflammatory conditions, the PTX3-content in plasma rises rapidly and dramatically to reach a maximum level of 200–800 ng/ml within 6 to 8 hours [7].

Along with ficolins and collectins, pentraxins recognise pathogen associated molecular patterns (PAMPs) and cooperate with the cellular arm of the innate immunity in activating and orientating the humoral immune response [7]. PTX3 binds several pathogens, including selected bacteria, fungi and viruses [4]. In this setting, it functions as an opsonising agent facilitating pathogen recognition [8].

Besides pathogens, PTX3 recognises and binds complement components, extracellular matrix, and growth factors. PTX3 appears to act as a modulator of the complement system as it is able to both cause activation and inhibition depending on the bound ligand [9]. Furthermore, the binding of extracellular matrix proteins, such as tumor necrosis factor-inducible gene 6 protein (TSG-6) and inter-alpha-trypsin inhibitor (IαI), along with the fibroblast growth factor FGF-2 has proven PTX3 to be involved in tissue remodelling, including the process of cumulus oophorus assembly, angiogenesis and restenosis [2]. Finally, PTX3 has been shown to bind late apoptotic cells, and in this way help the immune system to distinguish between self, modified self and non-self [10].

The systemic inflammatory response syndrome (SIRS) is a non-specific, inflammatory host response to a variety of insults. These can be both infectious and non-infectious, e.g. multiple trauma, ischemia and pancreatitis. When the SIRS criteria are met, and the cause of the symptoms confirmed or strongly suggested to have an infectious origin, the term sepsis is applied. Patients with sepsis are at risk of progressing into severe sepsis, and finally septic shock [11]. The mortality rate of SIRS, sepsis, severe sepsis and septic shock is high, ranging from approximately 10% in SIRS to 60% in septic shock [12]. While identification and treatment of sepsis has led to a decrease in mortality, the incidence continues to increase resulting in a still larger number of deaths annually [13]. Worldwide, sepsis is thus the major cause of death in intensive care units (ICU) and therefore an area of concern [14].

In order to improve the survival of patients with SIRS and sepsis, it is essential to identify the individuals at high risk. One approach for this identification is constantly evaluating reliable biological markers. Diagnostic markers should be able to accurately detect the disease early in its course while prognostic markers must predict the progression of the disease. Procalcitonin and CRP are biological markers currently in clinical use for detection of infection and unspecific inflammation, respectively, in the therapeutic management of SIRS and sepsis [15].

Recent studies have shown that PTX3 can be used as a marker for disease severity and fatal outcome in cardiovascular, inflammatory and infectious diseases [16]–[21]. Thus, we developed a novel assay for measurement of PTX3 in body fluids and examined the serum levels from patients with SIRS and sepsis admitted to an ICU as well as controls in order to review the validity of PTX3 as a biomarker in ICU patients. In addition we compared the performance of the PTX3 assay with the measurement of CRP in the same patient samples.

## Results

### Construction of the PTX3 ELISA

#### Sample dilution and administration

The patient serum samples (n = 261) were added in a dilution of 1∶5, as were the samples from the blood donors (n = 100). However, a number of patient samples (n = 160) exceeded the upper limit of quantification and were thus re-run in a higher dilution: 1∶20. Moreover, a number of samples, from both patients (n = 38) and controls (n = 31), exceeded the cut-off point on the control plates, as described previously. These samples were re-run in a sampling buffer with addition of CrossDown buffer and EDTA in attempt to remove the interfering antibodies disturbing the signal from the PTX3 detection. All samples were applied in duplicates, and the mean values were used in subsequent calculations.

#### Standard curve

The standard curve of the assay was constructed by applying a 2-fold serial dilution of the culture supernatant of the rPTX3 to every plate. The starting concentration of rPTX3 was set to 20 ng/ml and from that point diluted 6 or 8 times to a final concentration of 0.63 or 0.16 ng/ml. The mean optical densities (OD) and the corresponding concentrations were subjected to a logistic, nonlinear regression model regarding four parameters including minimum and maximum asymptote, inflection point and slope factor. This was expressed by a sigmoidal dose-response curve and enabled us to estimate concentrations from OD-values at both the bottom, the center and the top.

The sensitivity of the standard curve was assessed by including a negative control to the assay set-up. A cut-off was set at the OD-value of the negative control multiplied by the standard deviation times 3. Any sample with an OD-value below this cut-off value (0.3 ng/ml) was considered undetectable.

#### Construction of the assay calibrator

**Figure 1:**
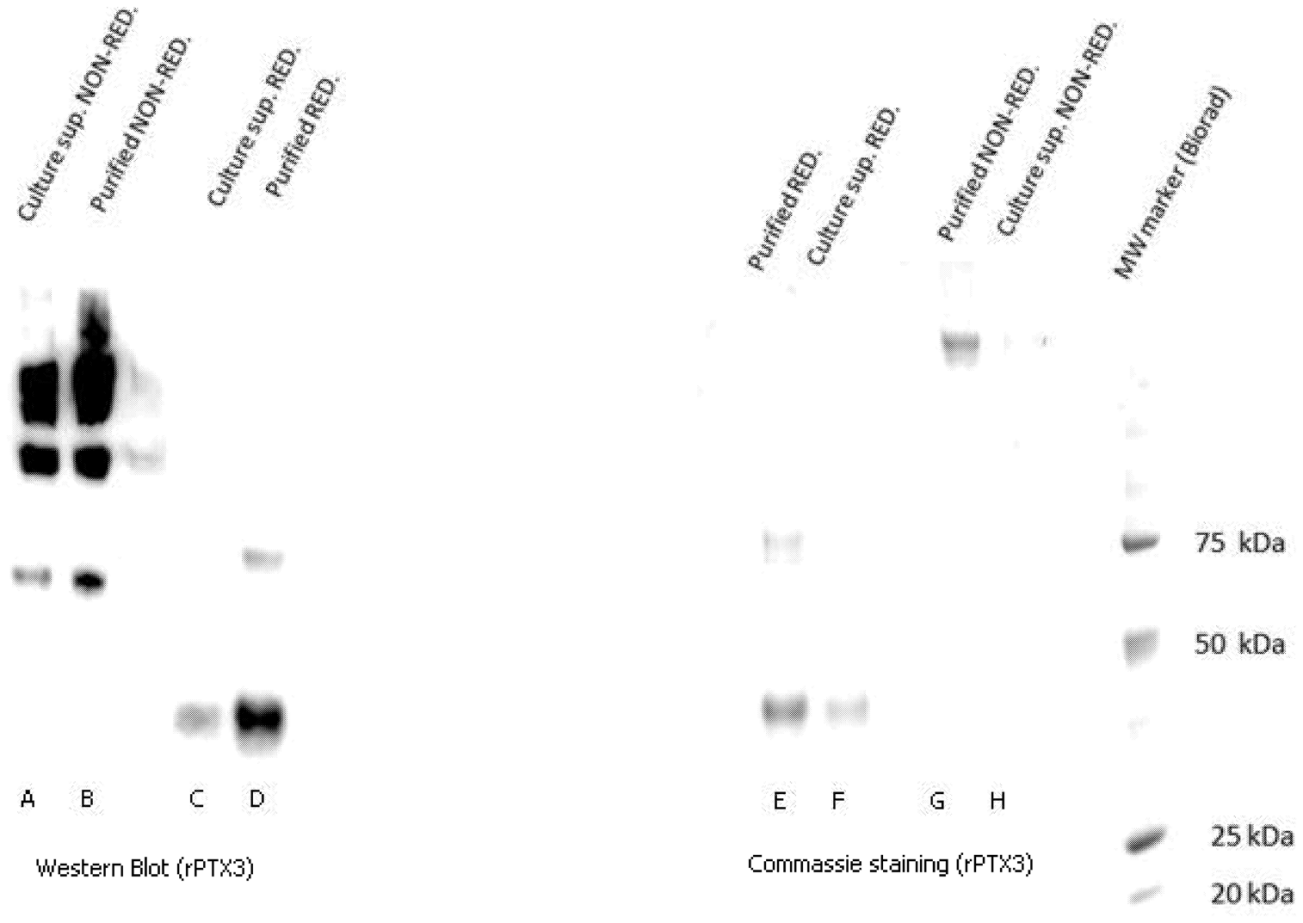
Western blot (left) and Commassie staining (right) of recombinant PTX3 are shown. Lanes A and B show the culture supernatant (A) and purified protein (B) under non-reducing conditions: step ladder formation reflecting the equilibrium between the different PTX3 oligomers. Lanes C, D, E and F show the culture supernatant (C, F) and the purified protein (D, E) under reducing conditions: PTX3 monomer at 45 kDa. Lanes G and H show the purified protein (G) and the culture supernatant (H) under non-reducing conditions: the predominantly multimer of PTX3 with a MW of 340 kDa. The rightmost lane provides the standard for molecular weight ranging from 10–250 kDa.

Initially, the rPTX3 was purified in order to create a calibrator for the ELISA system. The purification was evaluated by Coomassie staining and Western Blot (Figure 1).

Under reducing conditions, a lower band is apparent in both systems (lanes C, D, E, F). This band represents the major PTX3 monomer in that it corresponds to a molecular weight (MW) of 45 kDa [7].

In the Coomassie staining, only one band (>200 kDa) is visible in the column with the purified protein under nonreducing conditions (lane G). This probably reflects the predominantly multimer with an apparent molecular mass of 340 kDa [3].

Within the similar settings in the Western Blot, a step ladder pattern appears in both the column containing the purified protein and the one with the culture supernatant (lanes A, B). This pattern probably reflects the equilibrium existing between the different PTX3 oligomer forms [22].

In the first experiments, when assessing the stability of the immunoassay, the purified rPTX3 was applied and functioned as calibrator. However, after several months stored at −20°C the purified rPTX3 became unstable and was no longer detectable in our ELISA system. Thus the purified rPTX3 was replaced by the culture supernatant that was stored under the same conditions and did not show any alternations.

#### Assay stability

The stability of the immunoassay was assessed by determining the intra-assay (CV = 6.9%) and inter-assay variation (CV = 15.6%), as discussed previously. Moreover, the analytical recovery of a plasma sample from 9 consecutive freezing-thawing cycles was tested (CV = 3.7%) addressing that the patient samples have been thawed and refrozen a couple of times prior to use in the PTX3 assay.

Additionally, a serum sample from one healthy volunteer was used as a positive control and applied to every assay in order to evaluate the consistency of this value in each one.

#### Comparison with a commercial PTX3 ELISA kit

We investigated 26 paired samples in the present PTX3 ELISA and compared the results with a commercial PTX3 ELISA. The assays correlated highly significantly (rho 0.8, p<0.001) (data not shown).

### PTX3 levels in healthy controls and patients with SIRS and ROC curve analysis

**Figure 2:**
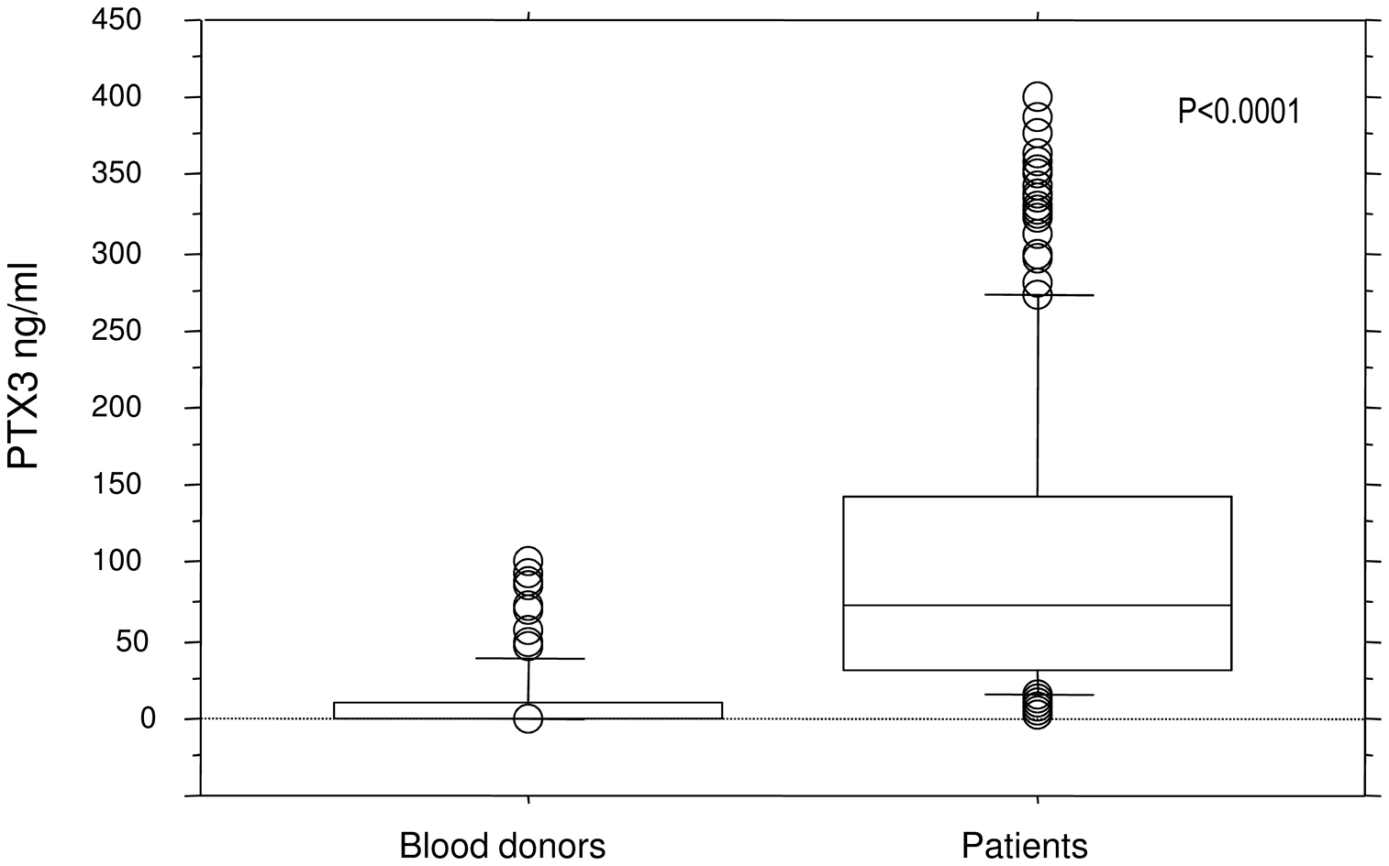
Serum levels of PTX3 in healthy controls (n = 100) (left) and patients with SIRS or sepsis (n = 261) (right) are shown. The PTX3 levels were elevated in the patients compared with the controls (Mann-Whitney, p<0.0001). The ranges (0–100 ng/ml in controls, 0.8–400 ng/ml in patients), the interquartile ranges (in controls 0.0–9.7 ng/ml and 31.1–143.3 ng/ml in patients) and the medians (0 ng/ml in controls and 71.3 ng/ml in patients) are indicated.

In 100 blood donors, the median PTX3 serum level was estimated to be 0.0 ng/ml, ranging from 0 ng/ml to 100 ng/ml, while the median PTX3 value in patients was estimated to be 71.3 ng/ml (Mann.Whitney: p<0.0001), spanning from 0.8 ng/ml to 400 ng/ml (Figure 2). The upper limit was arbitrarily set to 400 ng/ml as the PTX3 concentrations in some patients exceeded the standard curve limitation. Assessed by ROC analysis, serum concentrations of PTX3 at time of admission were sufficiently specific (85.0%) and sensitive (89.1%) to discriminate between healthy controls and SIRS (area under the curve (AUC) 0.922 (95% CI 0.892 to 0.946, p<0.0001). The optimal cut off value was found to be 16.0 ng/ml.

### Demographics of study population and prognostic value of PTX3

**Figure 3:**
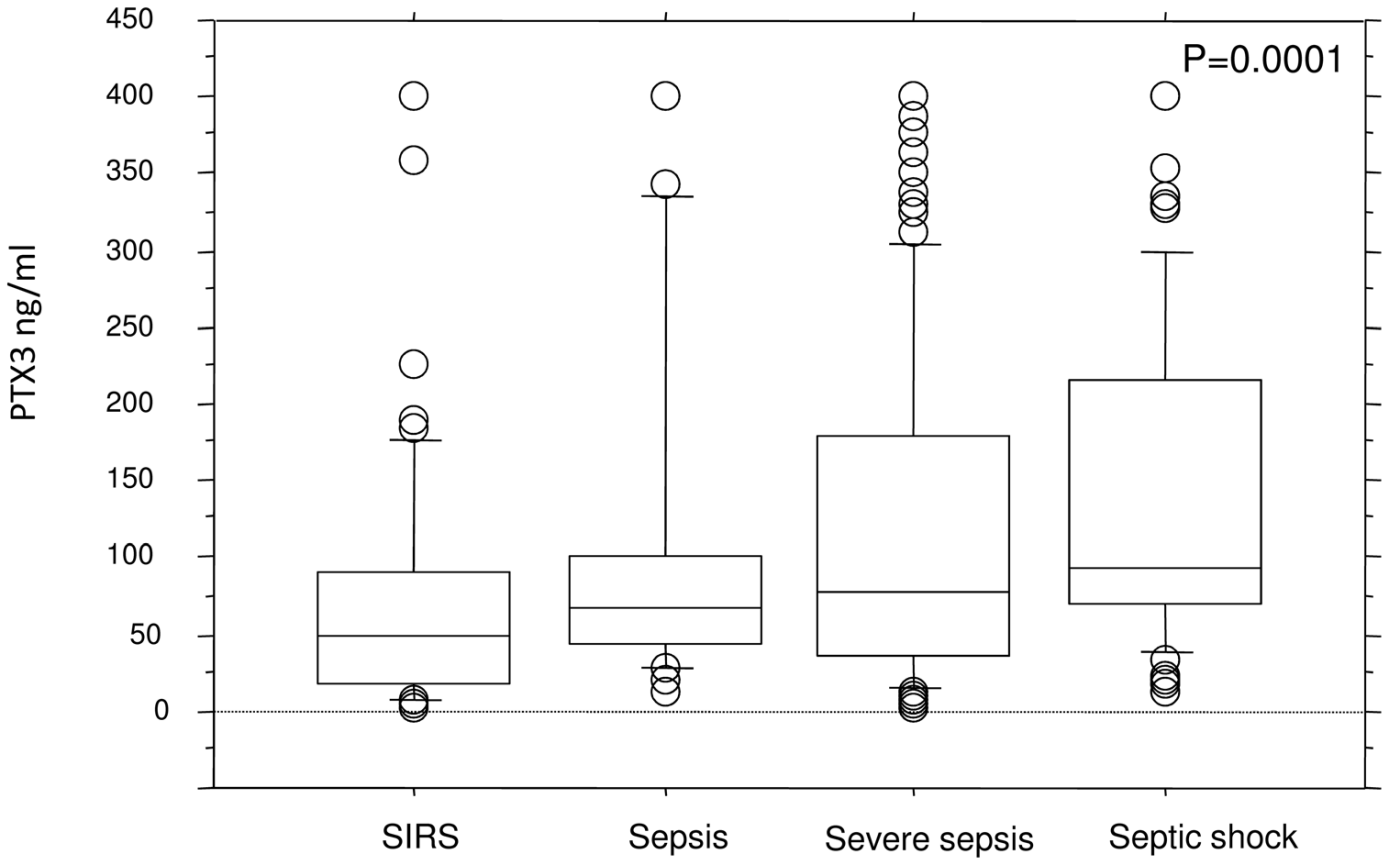
Serum levels of PTX3 in patients classified as having SIRS (n = 73), sepsis (n = 26), severe sepsis (n = 97) and septic shock (n = 65), (Kruskal-Wallis, p = 0.0001) are shown. Ranges, interquartile ranges and medians are indicated. Using a Dunn's post test revealed that only the SIRS group differed significantly from the other groups (p<0.05), while no significant difference was observed between the sepsis, severe sepsis and septic shock groups (p>0.05).

**Figure 4:**
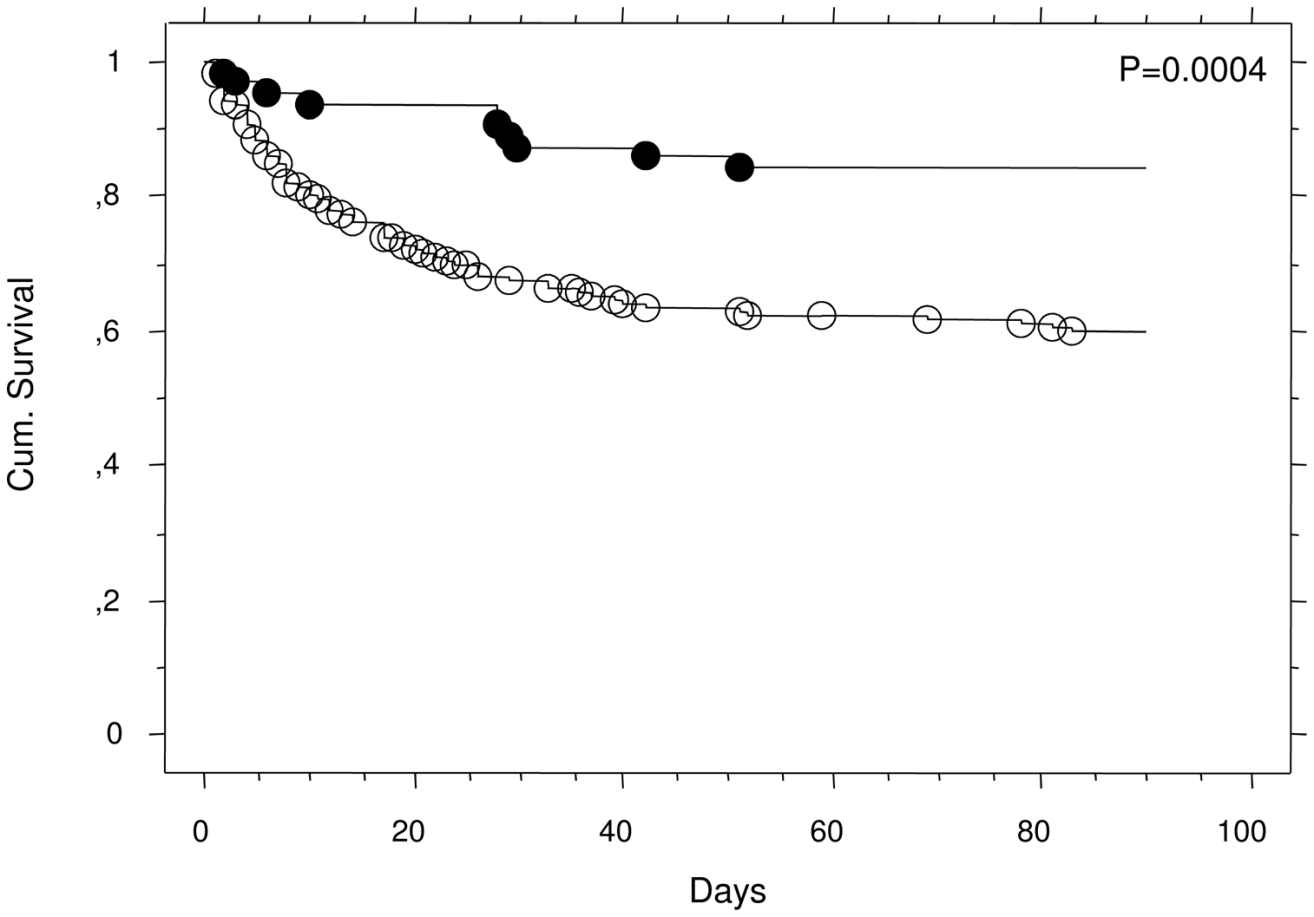
Kaplan-Meier plot of 90 day survival after admission to the ICU in patients with SIRS or sepsis is shown. Closed circles indicate PTX3 levels below the 25th percentile, while open circles indicate PTX3 levels above the 25th percentile (log rank, p = 0.0004). The 25th percentile corresponds to a PTX3 level of 32.6 ng/ml.

Of the 261 consecutive patients with SIRS with eligible samples enrolled, 45% were admitted to the ICU due to postoperative complications after acute or elective surgery, while 55% were admitted due to nonsurgical causes. The median age was 63 (range, 16–88) years and the sex distribution was 47% females and 53% males (Table 1). Serum concentrations of PTX3 did not differ between sexes nor by whether the patients were admitted with a surgical or nonsurgical diagnosis, but increased significantly with age (rho 0.27, p<0.0001) and disease severity as assessed by SAPS2 score (rho 0.28, p<0.0001) (Table 2). Increased serum concentrations of PTX3 were observed in the patients with a positive blood culture taken at admission (Mann-Whitney, p = 0.018) (Table 2). The PTX3 serum concentration was significantly lower in the patients classified having SIRS only compared with those patients classified as having sepsis (Mann Witney, p<0.0001) (Table 2). The median serum PTX3 concentration increased gradually when moving from SIRS without infection through sepsis, severe sepsis, and septic shock (Kruskall Wallis, p = 0.0001) (Figure 3 and Table 2). However, using a Dunn's post test revealed that only the SIRS group differed significantly from the other groups (p<0.05), while no significant difference was observed between the sepsis, severe sepsis and septic shock groups (p>0.05). Median follow-up time was 873 (range, 0–1458) days. A total of 143 patients (55%) died, of which 49 (19%) died in the ICU and 77 (30%) elsewhere in the hospital. There was a significant association between increasing levels of PTX3 and 90 day survival using univariate Cox regression analysis (chi square: 11.1, p = 0.0009), when adjusted for sex and age the association was still significant ( chi square: 5.57, p = 0.018). When we dichotomized the PTX3 values at the 25th percentile (≤32.6 versus >32.6 ng/ml), it was revealed once again that a high PTX3 level at admission to the ICU was associated with decreased 90 day survival (log rank test p = 0.0004) (Figure 4). Univariate Cox regression analysis revealed that a high PTX3 value above the 25th percentile was associated with a hazard ratio of 3.0 (p = 0.0009), which was still significant when adjusted for age and gender (hazard ratio 2.43, p = 0.0087) (Table 3). When we used ROC analysis to find the best discriminator between high and low levels in relation to survival, the best fit was found to be 39.3 ng/ml. Using this cut off value in a univariate Cox regression analysis a hazard ratio of 3.1 (p = 0.0003) was found and after adjustment for age and gender a hazard ratio of 2.52 (p = 0.003) was found.

**Table 1:** Demographics of the patients.

**Number of patients**	261
**Age, median years (range)**	63 (18–88)
**Female /male, no. (%)**	122 (47)/139 (53)
**Type of admission**	
Acute surgery, no. (%)	111 (42)
Elective surgery, no. (%)	8 (3)
Medical, no. (%)	142 (55)
**SAPS2 score, median (range)**	36 (6–89)
**Disease severity**	
SIRS, no. (%)	73 (28)
Sepsis, no. (%)	26 (10)
Severe sepsis, no. (%)	97 (37)
Septic shock, no. (%)	65 (25)

**Table 2:** Serum concentrations of PTX3 according to baseline covariates.

			PTX3 (ng/ml)			
Covariate	N (%)		Mean ± SEM		Median (IQR)	P
**Sex**						0.81
Female	122 (47)		115±10		72 (124)	
Male	139 (53)		108±8		79 (107)	
**Type of admission**						0.88
Medical	142 (54)		117±10		71 (129)	
Surgical	119 (46)		104±9		79 (99)	
**Culture positive**						0.018
Yes	129 (49)		128±10		77 (166)	
No	132 (51)		95±8		70 (84)	
**SIRS vs all sepsis**						<0.0001
SIRS	73 (27)		76±1050 (72)			
All sepsis	188 (73)		125±881 (152)			
**Disease severity**						0.0001
SIRS	73 (27)		76±10		50 (72)	
Sepsis	26 (10)		113±23		66 (55)	
Severe sepsis	97 (37)		118±11		77 (141)	
Septic shock	65 (25)		139±13		93 (145)	
**Correlation analysis**						
**PTX3 (ng/ml) versus**		**Spearman's rank rho**		**P**		
Age		0.27		<0.0001		
SAPS2 score		0.28		<0.0001		

SEM, standard error of the mean; SIRS, systemic inflammatory response syndrome; SAPS2, simplified acute physiology score. All sepsis means sepsis, severe sepsis and septic shock.

**Table 3:** Cox regression analyses of association between serum concentrations of PTX3 below and above the 25th percentile at time of admission and 90-day survival.

Analysis/Covariate	Hazard ratio	95% CI	p
**Univariate**			
Serum PTX3 level, ≤32.6 vs.>32.6 ng/ml	3.0	1.57–5.89	0.0009
**Multivariate**			
Serum PTX3 level, ≤32.6 vs.>32.6 ng/ml	2.43	1.25–4.7	0.0087
Age	1.033	1.01–1.05	<0.0001
Gender, female vs. male	0.94	0.61–1.42	0.77

CI, confidence interval.

### PTX3 versus CRP

In order to compare the performance of PTX3 with an established biomarker in SIRS and sepsis we compared PTX3 with CRP in the patients. The levels of PTX3 and CRP correlated significantly (rho 0.35, p<0.0001). Using ROC curve analysis to show performance in order to discriminate between SIRS and sepsis no overall significant difference was observed (p = 0.56) The CRP levels also correlated significantly with the SAPS2 score (rho 132, p = 0.0329). However, in contrast to PTX3 CRP could not be used to predict 90 days fatal outcome using linear univariate Cox regression analysis (chi-square 2.488, p = 0.11) or when the analysis where adjusted for age and gender (chi-square: 1.04, p = 0.3079). When performing a Cox regression analysis taking both PTX3 and CRP into account PTX3 was significant (chi square: 9,289, p = 0.0023), while CRP was not (chi square: 0.739, p = 0.389). This was also the case when we included age and gender in the model: PTX3 (chi square: 4.68, p = 0.03) and CRP (chi square: 0.187, p = 0.665).

## Discussion

PTX3 are barely detectable in human plasma and serum under normal conditions, and although dramatically increased during inflammation, the upper range of PTX3 in plasma and serum does not exceed a concentration of 800 ng/ml. Thus, it was necessary to develop an immunoassay with high sensitivity in order to detect PTX3 for clinical use. Highly specific monoclonal antibodies against human PTX3 were produced and used to develop the new sandwich ELISA system. With this, we were able to detect PTX3 in serum at concentrations down to 0.3 ng/ml. Moreover, the established assay was very stable and capable of measuring PTX3 exposed to consecutive freezing thawing cycles. Thus, this assay may also be applied on blood samples that have been stored over longer periods of time.

In addition to the high sensitivity, a high specificity is required to ensure that other molecules are not interfering with the detection of PTX3. These demands implied a number of complications in establishment of the ELISA system. When dealing with a low dilution factor (1∶5), a large amount of molecules will be present in plasma and serum. In analysing our measurements, we discovered the need for an addition of molecules blocking interfering antibodies in order to obtain reliable results.

A strength of this new assay was the very high correlation with a commercially available PTX3 assay, suggesting that the results obtained with this assay may be comparable with other studies investigating PTX3. In addition, this assay has properties in line with PTX3 assays applied in previous studies concerning both stability and sensitivity – qualifying it as a suitable approach for the continuous evaluation of PTX3 as a potential biomarker in clinical settings. The validity of this new assay was shown when we investigated the use of PTX3 as biomarker in patients with SIRS and sepsis compared with controls, which consisted of randomly selected Danish blood donors. Comparison of these groups showed that PTX3 is a good at discriminating healthy controls from patients with SIRS and sepsis since the ROC analysis showed that the assay was highly specific and sensitive. Elevated levels of PTX3 were observed in the patients (median: 71.3 ng/ml) compared with controls (median: 0.0 ng/ml), which is consistent with recent findings [18]–[20]. Moreover, PTX3 was shown to be increased with increasing disease severity from SIRS to sepsis. The level of PTX3 also correlated with the SAPS2 severity score. Patients with high levels of PTX3 were at increased risk of fatal outcome as judged by the 90 day mortality rate. This was still the case after adjusting for age and gender as possible confounders. Both the use of 25th percentile and the use of ROC analysis to dichotomize the patients to find the best discriminator between high and low levels of PTX3 gave very similar results.

Huttunen et al. [19] have assessed the potential of PTX3 as a marker in bacteremic patients while evaluating other established biomarkers as well. This study showed that PTX3 had a better prognostic value than CRP. We also tested for CRP values in the patients included in this study and found that PTX3 and CRP correlated significantly and had similar performance in discriminating between SIRS and sepsis, but that PTX3 could predict 90 days fatal outcome, while CRP could not when tested in Cox regression analysis.

Some of the controls did in fact have increased PTX3 levels. Whether this indicates that these controls indeed had an ongoing subclinical inflammatory process is possible and raises the possibility that PTX3 might be a potential future risk biomarker in diseases characterized by subclinical inflammation as cardiovascular diseases even in individuals with no apparent symptoms in line with what has been suggested for CRP, suPAR and YKL-40 [23]–[25].

Certain weaknesses of this study should be considered. It is a single center study and we allowed only patients that did survive at least 6 hours at the ICU to be included. Moreover, only one measurement from each patient was performed. Nevertheless, the results obtained appeared to be able to discriminate very well between patients and controls, to some degrees reflect disease severity and maybe more important to be of prognostic value. In future studies repetitive measurements should be performed and also comprehensive measurements of other potential biomarkers other than CRP in order to better define algorithms to monitor ICU patients.

In conclusion, in this study, we developed a highly sensitive PTX3 ELISA in order to investigate and extend the finding that PTX3 levels are associated with disease severity and mortality in infectious and inflammatory disorders. PTX3 values proved to be significantly elevated in patients with SIRS and sepsis compared to healthy controls. In addition, PTX3 levels in patients at admission to the ICU correlated with disease severity while patients with the lowest concentrations were shown to have better prognosis than the ones with higher levels. Thus, our results support recent findings on PTX3 in infectious disorders and thereby emphasize the potential of PTX3 as an important prognostic marker in ICU patients.

## Materials and Methods

### Patients

The patients in this cohort have previously been studied [26], [27] and consist of 272 consecutive adult patients (>18 years) with SIRS admitted to the ICU at Glostrup University Hospital, Copenhagen, Denmark over a period of 18 months. Patients were assessed for the Simplified Acute Physiology Score 2 (SAPS2) [28] within 24 hours. Patients were included in the study if they survived at least 6 hours in the ICU and met the criteria for SIRS according to Bone et al. [29]. Exclusion criteria were a neutrophil count <1.0×10^9^ cells/l before onset of sepsis, infection associated with burns, suspected or documented recent acute myocardial infarction, or lack of commitment to full life-support measures by the primary physician. Peripheral blood samples for serum were obtained immediately after admission to the ICU. Two hundred and seventy-two patients were included, of which 261 serum samples stored at −80°C were eligible for this study.

Serum samples from 100 blood donors served as controls for this study.

### Ethics statement

The study was approved by the local ethics committee: The Research Ethics Committee of the County of Copenhagen. Written informed consent approved by the Local Ethics Committee was obtained from all patients or from their nearest relatives. Follow-up registration of death was provided by the Danish Central Office of Civil Registration.

### Preparation of recombinant PTX3

The cDNA encoding for the PTX3 full length (1311-bp) was sub-cloned in pED vector by standard cloning techniques and transfected to Chinese hamster ovary (CHO DG44) cells in the presence of Lipofectamine as described previously [30]. Culture supernatants from stable transfected CHO cells were harvested.

### Generation of monoclonal antibodies

BALB/c×NMRI mice were immunised subcutaneously two times with 25 µg recombinant human PTX3 (rPTX3) purchased from R&D systems (Cat. No. 1826-TS) adsorbed to Al(OH)_3_, mixed in 1∶1 ratio with Freud's incomplete adjuvant. Four days prior to the fusion, the mice received an intravenous injection with 25 µg antigen administered with adrenalin. The fusion was done essentially as described by Köhler and Milstein [31]. The SP2/0-AG14 myeloma cell line was used as a fusion partner. The selection of clones was based on their ability to bind rPTX3 captured by a polyclonal goat anti human PTX3 antibody purchased from R&D systems (Cat. No. AF1826) in an ELISA system.

### Purification and biotinylation of antibodies

Monoclonal antibodies from 11 selected clones were tested in ELISA using a rabbit anti-mouse detecting antibody (Dako Z0259, Lot 065). 10 out of 11 monoclonal antibodies were chosen and therefore purified in a small quantity by protein A affinity chromatography.

Clones aPTX3-20 and aPTX3-73 were subsequently purified in a larger scale using Protein A affinity chromatography by applying it to HiTrap^TM^ rProtein A FF columns (GE Healthcare BioSciences) according to the manufacturer's instructions. They we determined to be of the IgG1kappa immunoglobulin subtype.

Subsequently, all of the purified antibodies were biotinylated using ImmunProbe™ Biotinylation Kit (BK-101, Sigma-Aldrich) according to the manufacturer's guidelines.

### Purification of recombinant proteins

The culture supernatant of the rPTX3 produced in our laboratory was purified by immunochromatography. A pool of the monoclonal antibodies was linked to Cyanogen Bromide-activated Sepharose^TM^ 4 Fast Flow (Prod. No. 17-0981-01, GE Healthcare Life Sciences) and subsequently transferred to an affinity column. The culture supernatant was run through the column overnight at 4°C, washed and following eluted with 0.5% citric acid. To neutralise the eluates, 1 M Tris buffer (pH 10) was added, and subsequently the eluates were dialysed. The purification was evaluated by SDS-page and immunoblotting, as described below.

### Construction of the PTX3 sandwich ELISA

The PTX3 assay was constructed as a sandwich ELISA based on the principle of antigen recognition initially by a solid phase antibody and secondly by an enzyme-linked antibody. These enable the capturing of the protein and the detection of the antibody-antigen complex, respectively.

Initially, epitope mapping was performed in ELISA testing all possible combinations of the 10 purified anti-PTX3 monoclonal antibodies. Each combination was evaluated according to the sensitivity of their detection of rPTX3. Clone aPTX3-20 was found to be the optimal coating antibody while both clones aPTX3-66 and aPTX3-73 qualified as detection antibodies. While assessing the ability of PTX3 detection by these antibodies in body fluids, both separately and combined, aPTX3-73 turned out to be the preferred one. Furthermore, the sensitivity was not increased, when aPTX3-66 were added, pointing to the fact that the detection antibodies may share the same determinant.

In order to optimise the sandwich ELISA system fully, a chessboard titration of aPTX3-20 and aPTX3-73 in different concentrations was performed in order to determine the optimum concentrations.

### Experimental procedure

The ELISA was performed on microtiter plates (Nunc Immuno Plates, F96 Maxisorp). The working volume was 100 µl in all steps except for the last one where only 50 µl was applied. Between every step, the plates were washed four times in phosphate buffered saline +0.05% Tween-20 (PBS-T). All procedures were performed at room temperature, except for the initial coating.

At first, microtiter plates were coated with 0.5 µg/ml aPTX3-20 diluted in PBS by incubation overnight at 4°C.

The samples were applied along with a calibrator, a positive and a negative control diluted in a sampling buffer (see below), and were incubated for 2 hours.

The primary antibody, aPTX3-73, was added to the wells in a final concentration of 2 µg/ml diluted with PBS-T. The plates were left for 1 hour and 30 minutes incubation. A steptavidine-horseradish peroxidase (HRP-strep) conjugate (LOT 4646544, GE Healthcare) was added in a dilution of 1∶1500 with PBS-T and incubated for 45 minutes.

Subsequently, TMB-sens (Cat. No. 4850, Kem-En-Tech Diagnostics) was applied to the wells and developed in the dark for 8–10 minutes for signal achievement. At this point, a stop solution of 1 M sulphuric acid (H_2_SO_4_) was added.

Within 15 minutes after stopping the reaction, the optical density of each well was measured at 450 nm with an ELISA reader.

### Determination of PTX3 concentration in serum

The PTX3-content in the serum samples was assessed by correlating sample duplicates to a standard curve based on a 2-fold serial dilution of rPTX3 with known concentration. Patient samples were applied in a dilution of 1∶5 or 1∶20, while control samples were run solely in 1∶5.

### Design of sampling buffer and control assay

Human serum may contain endogenous antibodies that can interfere with the immunoassay, especially when dealing with a sandwich format. These antibodies include anti-animal antibodies, in particular human anti-mouse, auto-antibodies, and heterophilic antibodies. The last mentioned are polyreactive antibodies against poorly defined or even unknown antigens. In addition, our monoclonal antibodies were grown in medium containing fetal calf serum and might comprise remaining bovine proteins.

To ensure the specificity of the immunoassay, the sampling buffer was provided with 1% mouse-serum, 1% cow-serum, 2.5% CrossDown buffer (Prod. No. A6485, AppliChem) and 5 mM EDTA. While the sera and CrossDown help saturate the excessive binding sites, EDTA breaks all calcium-depending interactions causing PTX3 release from any calcium-dependent complex formation.

In order to eradicate erroneous results, all samples were run on a control plate coated with mouse immunoglobulins without specificity towards human proteins. A cut-off value for the optical density on the control plate was determined – resulting in exclusion of all samples with values above this point.

### Intra- and inter-assay variation

The intra-assay variation was determined by applying the same plasma sample in duplicates 10 times in the same assay, while the inter-assay variation was achieved by analysing the duplicates in 10 separate assays. The plasma sample applied was drawn from a healthy volunteer and subsequently exogenously rPTX3 was added. To quantify the extent of variability observed in the various assays, the coefficient of variation (CV) was determined. The CV represents the fraction of the standard deviation of the duplicate in relation to the mean value of the duplicate. Thus, the mean values and standard deviation of all the duplicates were calculated.

### Commercial human pentraxin 3 ELISA kit

To evaluate our ELISA, it was compared to a commercially available kit. The PTX3 sandwich ELISA kit (HK347) was purchased from Hycult Biotech. Randomly selected serum samples from both patients and controls, were run on the kit according to the manufacturer's instructions.

### SDS-PAGE and immunoblotting

Samples were loaded on a 4–12% (w/v) Bis-Tris polyacrylamide gel under both reducing and nonreducing conditions using the NuPAGE® system (Invitrogen) as recommended by the manufacturer. Subsequently, the gel was blotted to nitrocellulose (Hybond) using the Xcell II™ Blot Modul in NuPAGE® transfer buffer. With the antigens fixed on the membrane, residual binding sites were blocked with 5% skim milk.

In order to detect the proteins on the membrane, a pool of biotinylated antibodies, aPTX3-51 and aPTX3-73, was applied. HRP-strep was added in a dilution of 1∶5000 in PBS-T, and subsequently the membrane was washed extensively with PBS-T.

Finally, the membrane was developed with SuperSignal West Femto (Maximum Sensitive Substrate) on autoradiographic film. All Blue (Biorad) was used as standard for molecular weight.

### Determination of CRP concentration in serum

To review the potential of PTX3 as a novel biomarker in comparison with an already established one, we acquired a CRP Human ELISA Kit (Cat. No. KA0238, Abnova) and performed CRP measurements on all of the patient sera according to the manufacturer's guidelines.

### Statistics

The differences between the PTX3 levels in patients, in between patients and controls were assessed with respect to the medians using Mann-Whitney non-parametric test as well as receiver operating curve (ROC) analysis. Correlations were assessed by non-parametric Spearman rank analysis. Kruskal-Wallis analysis for unpaired group comparison was used to evaluate the effect of PTX3 level on disease severity and to establish differences between groups a Dunn's post test was performed. To estimate 90 day mortality Cox regression analysis and log rank test were performed. Kaplan-Meier survival analysis was applied on the patients with the 25% lowest PTX3 concentration in comparison with the rest. Two-tailed P-values were calculated throughout.
